# Visualising the endothelial glycocalyx in dogs

**DOI:** 10.1016/j.tvjl.2022.105844

**Published:** 2022-07

**Authors:** Sara J. Lawrence-Mills, Chris R. Neal, Simon C. Satchell, Gavin I. Welsh, Rebecca R. Foster, Natalie Finch

**Affiliations:** a Bristol Renal, Bristol Medical School, University of Bristol, Bristol, UK; b The Royal Veterinary College, University of London, North Mymms, UK; c Bristol Veterinary School, University of Bristol, Langford, UK; d Langford Vets, Langford House, Langford, UK

**Keywords:** Alcian blue, Canine, Microvasculature, Transmission electron microscopy

## Abstract

The endothelial glycocalyx (eGlx) lines the luminal surface of endothelial cells. It is critical in maintaining vascular health and when damaged contributes to many diseases. Its fragility makes studying the eGlx technically challenging. The current reference standard for eGlx visualisation, by electron microscopy using glutaraldehyde/Alcian blue perfusion fixation, has not been previously reported in dogs. Established techniques were applied to achieve visualisation of the eGlx in the microvasculature of reproductive tissue in five healthy dogs undergoing elective neutering. Uterine and testicular artery samples underwent perfusion fixation, in the presence of Alcian blue, prior to transmission electron microscopy imaging. Image processing software was used to determine eGlx depth. EGlx was visualised in the arteries of two dogs, one testicular and one uterine, with median (range) eGlx depths of 68.2 nm (32.1–122.9 nm) and 47.6 nm (26.1–129.4 nm) respectively. Study of the eGlx is technically challenging, particularly its direct visualisation in clinical samples. Further research is needed to develop more clinically applicable techniques to measure eGlx health.

## Highlights

• Canine glycocalyx has not previously been visualised using the reference technique.
• The endothelial glycocalyx was visualised in dog uterine and testicular arteries.
• Direct visualisation of the endothelial glycocalyx was technically challenging.

The endothelial glycocalyx (eGlx) is a gel-like matrix composed of proteins and glycosaminoglycans (GAGs; Hegermann et al., 2016; Ushiyama et al., 2016) covering the luminal surface of vascular endothelial cells. The eGlx has critical functions including regulation of vascular permeability (Ushiyama et al., 2016) and resistance of spontaneous coagulation (Broekhuizen et al., 2009). It is critical in maintaining vascular health (Alphonsus and Rodseth, 2014), and eGlx dysfunction is implicated in a plethora of disease processes in different species (Ueno et al., 2004, Salmon et al., 2012, Kolářová et al., 2014, Lawrence-Mills et al., 2022, In Press). Moreover, the eGlx offers an important therapeutic target (Broekhuizen et al., 2009).

The main components of the eGlx are proteoglycans and glycoproteins, comprising various core proteins and GAG side chains, free GAGs, such as hyaluronan, and soluble plasma proteins (Reitsma et al., 2007). The fragility of the eGlx makes visualisation and monitoring technically challenging (Reitsma et al., 2007, Chevalier et al., 2017). The reference standard technique for eGlx visualisation uses perfusion of cations such as Alcian blue (Hegermann et al., 2016) that bind negatively charged sulfated GAGs in the eGlx (Curran et al., 1965) to enable direct visualisation using transmission electron microscopy (TEM). Visualisation of the eGlx in dogs using this reference standard is lacking in published literature and yet is essential for advancing research in this field. The aim of this project was to visualise the eGlx in healthy dogs using Alcian blue perfusion.

**Fig. 1:**
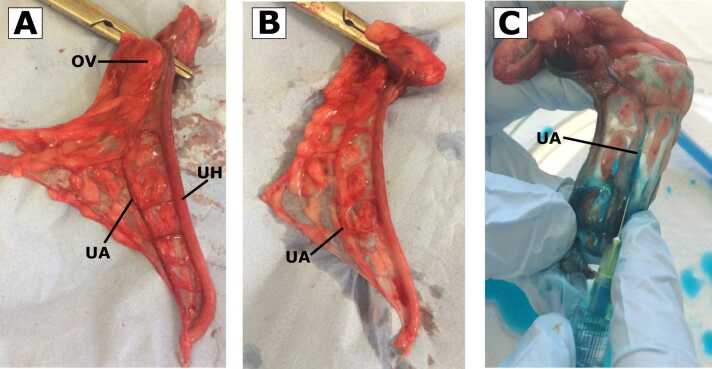
These images depict the process of perfusing a uterine artery sample prior to processing for TEM. A) Uterine horn with uterine artery labelled. B) Following perfusion with glucose-free mammalian Ringer-Locke’s solution to remove plasma proteins and red blood cells. C) After perfusion with Alcian blue fixative solution. OV, ovary; UA, uterine artery; UH, uterine horn.

**Fig. 2:**
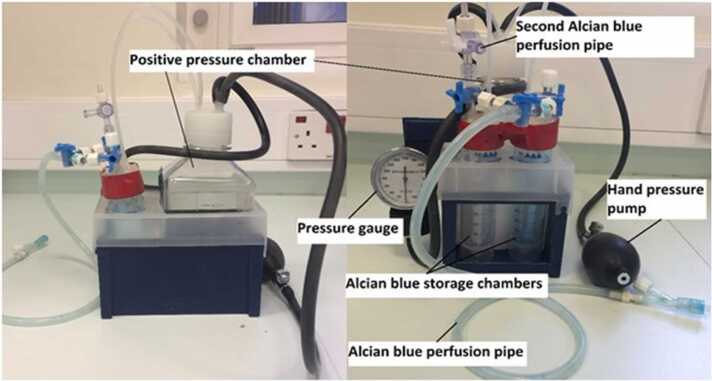
Front and side views of the equipment used to achieve Alcian blue perfusion and fixation for endothelial glycocalyx visualisation. Two 50 ml Falcon tube reservoirs were used as Alcian blue storage chambers (one flush, one fixative). These were attached via taps to a Y connector and a catheter. Controlled perfusion pressure was applied via an air pressure reservoir, the positive pressure chamber comprising a 500 ml plastic bottle maintained at 100 mmHg by a sphygmomanometer bulb. The reservoir taps allowed selection of either flush or fix reservoir via the Alcian blue perfusion pipes to the catheter.

Testicular and uterine tissue was prospectively collected during elective neutering of client-owned dogs at Langford Vets, University of Bristol. Health status was confirmed via a detailed history and physical examination. Informed owner consent was obtained. The study was approved by the University of Bristol Animal Welfare and Ethical Review Body (Approval number, VIN/16/047; Approval date, 24 November 2016). The anaesthetic protocol used is outlined in the Supplementary material. The eGlx visualisation technique was based on a methodology utilised in mice (Oltean et al., 2015) and rats (Desideri et al., 2018). The respective uterine or testicular artery was cannulated and flushed with compound sodium lactate at a standardised pressure of 100 mmHg within 2 min of clamping for a minimum of 25 s. Tissues were then flushed with glucose-free mammalian Ringer-Locke’s solution (see Supplementary material) at a continuous pressure of 100 mmHg for a minimum of 25 s or until visual confirmation of the removal of plasma proteins and red blood cells (Fig. 1), before perfusion with Alcian blue fixative (0.1 % Alcian blue and 1 % glutaraldehyde in mammalian Ringer-Locke’s solution) using a purpose-built perfusion kit (Fig. 2). Tissues were sectioned into 1 mm cubes and stored in fixative solution (0.1 % Alcian blue and 2.5 % glutaraldehyde in 0.1 M sodium cacodylate) at 4 °C until TEM processing. Samples underwent standard processing for TEM (see Supplementary material). Six TEM images per dog were analysed using image processing software (FIJI^1^), with a minimum of 10 eGlx measurements obtained per image at random locations along the endothelium (see Supplementary material). The median (range) eGlx depth was determined for each dog.

**Fig. 3:**
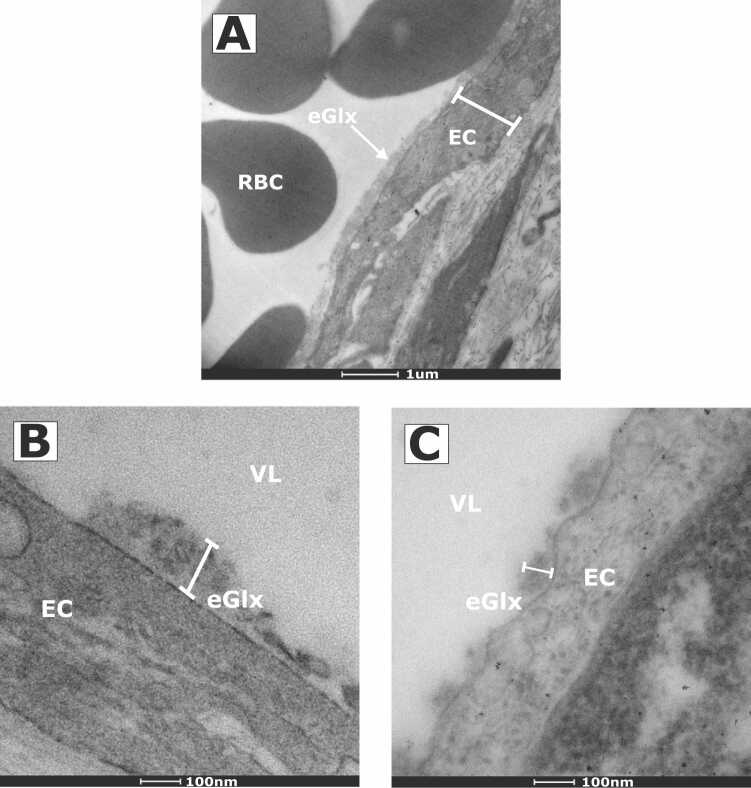
Transmission electron microscopy images of dog endothelial glycocalyx. A) Lower and B) Higher power images of dog uterine artery (NB: different sections are shown). C) Higher power image of dog testicular artery. EC, endothelial cell; eGlx, endothelial glycocalyx; RBC, red blood cell; VL, vessel lumen.

Testicular tissue was collected from four dogs and uterine tissue from one. Median age was 8 months (range, 7–24 months). Breeds included two Yorkshire terriers, one Cocker spaniel, and two Crossbreed dogs. The eGlx was successfully visualised in two dogs (Fig. 3), one male and one female, both Yorkshire terriers. Successful visualisation was achieved in the last two samples collected. The median (range) eGlx depth in testicular and uterine arteries was 68.2 nm (32.1–122.9 nm) and 47.6 nm (26.1–129.4 nm), respectively.

This study achieved eGlx visualisation, using the reference standard method of electron microscopy following Alcian blue perfusion, for the first time in dogs. The technical difficulties of direct eGlx visualisation are apparent with eGlx identified in only two of the five samples collected. Successful visualisation in the latter samples, suggested a degree of improvement with technique over time. This learning element should be considered in future research. Few studies in people and animal models have cited a percentage of successful visualisation; however, the difficulty in optimising conditions for Alcian blue perfusion is recognised (Chevalier et al., 2017, Mukai et al., 2020). Limitations of this technique include difficulties in effective flushing of plasma proteins and cellular debris, which may contribute to inadequate fixation and staining. Fig. 3 demonstrates incomplete removal of red blood cells. Further, in this study there was an unavoidable time lag between sample collection and flushing, although this was minimised as much as practically possible, there remains potential for plasma proteins to have degraded the eGlx (Jacob et al., 2007, Ebong et al., 2011).

The eGlx depths determined in the present study are similar to measurements obtained in mice (30–80 nm; Mukai et al., 2020) and rats (40–60 nm; Desideri et al., 2018) using Alcian blue perfusion. However, there is reported variability in eGlx thickness (Hegermann et al., 2016) influenced by sample handling, fixation, and processing (Reitsma et al., 2007, Kubaski et al., 2016). The heterogeneity of the eGlx is another challenging factor with unique differences reported across different vascular beds, organs, and species (Schulte and Spicer, 1983, Fernández-Sarmiento et al., 2020). Human studies have also identified sex-linked differences in eGlx depth (Brands et al., 2020). Further studies, including greater numbers of dogs, are needed to understand the influence of breed, sex, and neuter status on eGlx depth. For this multitude of reasons, it is difficult to directly compare eGlx measurements between studies (Hegermann et al., 2016).

The challenges of direct visualisation has led to the development of less invasive, indirect methods of eGlx evaluation (Nieuwdorp et al., 2008, Constantinescu et al., 2011) such as measurement of circulating eGlx components measured in humans including syndecans, hyaluronan and chondroitin sulphate (Padberg et al., 2014). Their use has been reported in naturally occurring diseases in dogs (Lawrence-Mills et al., 2022, Shaw et al., 2021). Other indirect methods of eGlx measurement include sidestream dark field imaging (Martens et al., 2013), where eGlx depth is inferred. This technique has been successfully implemented in humans (Pouska et al., 2018), cats (Yozova et al., 2021), dogs (Londoño et al., 2018) and horses (Mansour et al., 2021).

This study is the first to visualise the eGlx in dogs using the reference standard technique of Alcian blue perfusion. The specific challenges associated with direct eGlx visualisation in clinical samples are highlighted. Future studies should explore indirect measurements of eGlx health with direct visualisation measurements. Further research is needed to investigate the potential relationship between eGlx health and disease in veterinary species.

## Conflict of interest statement

MSD provided a financial award to Sara Lawrence-Mills (nee Hillyer) to support her presentation of the research at the British Small Animal Veterinary Association Congress 2018 and the Southern European Veterinary Conference 2018. None of the authors has any other financial or personal relationships that could inappropriately influence or bias the content of the paper.

## Footnotes

1 See: Image J Docs Downloads. https://imagej.net/Downloads (Accessed 25 May, 2022).
