# Supplementary material for: Visualising the endothelial glycocalyx in dogs
Source: Vet J. 2022 Jul;285:105844. doi: 10.1016/j.tvjl.2022.105844 (PMC9587350; doi:10.1016/j.tvjl.2022.105844)
Supplement: Supplementary file 1 — Supplementary material [file mmc1.docx]

**YTVJL-D-21-00527 Supplementary material**

*Anaesthetic protocol*

Dog castration protocol: Premedication with intravenous 5-10 μg/kg medetomidine and 0.02 mg/kg buprenorphine. Induction of anaesthesia with intravenous propofol to effect via a pre-placed intravenous catheter. Endotracheal intubation and maintenance of anaesthesia with inhalational isoflurane (percentage adjusted as required) in 100% oxygen. No perioperative fluid therapy.

Bitch spay protocol: Premedication with intravenous 5-10 μg/kg medetomidine and 0.3 mg/kg methadone. Induction of anaesthesia with intravenous propofol to effect via a pre-placed intravenous catheter. Endotracheal intubation and maintenance of anaesthesia with isoflurane (percentage adjusted as required) in 100% oxygen. No perioperative fluid therapy.

*Stock solutions*

1. Ringer-Locke’s stock solution X5 (x5 concentrate); 19.3 g NaCl, 0.86 g KCl, 0.74 g MgCl_2_.6.H_2_O, and 0.71 g CaCl_2_.2.H_2_O in distilled water to a total volume of 500 mL
2. 10.08% sucrose; 10.08 g sucrose in distilled water to a total volume of 100 mL.
3. Glucose free mammalian Ringer-Locke’s solution (x2 concentrate) (GFMRL2); 100 mL of Ringer-Locke’s stock solution X5, 0.365 g HEPES acid, and 0.250 g HEPES base in distilled water to a total volume of 250 mL.

*Working solutions*

1. Flush solution (*mammalian Ringer-Locke’s solution plus 4% sucrose*): 50 mL GFMRL2 and 40 mL 10.08% sucrose in distilled water to a total volume of 100 mL.
2. Perfusion fixative solution (*1.0% glutaraldehyde and 0.1% Alcian Blue in mammalian Ringer-Locke’s solution*; made up fresh immediately prior to use): 200 mL GFMRL2, 0.4 g Alcian blue 8GX (Sigma-Aldrich), 20 mL of 25% glutaraldehyde in distilled water to a total volume of 400 mL.
3. Immersion fixative solution (*2.5% glutaraldehyde and 0.1% Alcian Blue in 0.1M cacodylate buffer*; made up fresh immediately prior to use): 0.1g Alcian blue 8GX, 10 mL of 25% glutaraldehyde, 50 mL 0.2M sodium cacodylate buffer in distilled water to a total volume of 100 mL.

*Processing for transmission electron microscopy*

Samples were post-fixed in 1% osmium tetroxide, *en bloc* stained with 3% uranyl acetate, followed by ethanol dehydration and embedding in TAAB 812 resin mix (Agar Scientific). Sections were cut at 50-100 nm using an ultramicrotome and stained with 3% aqueous uranyl acetate and Reynolds' lead citrate stain. Electron micrographs were acquired using a Technai T12 electron microscope (Thermofisher Scientific, UK).

*Randomisation of measurement analysis*

Randomised measurements were achieved using a grid of 20,000 points per inches^2^, measurements were taken where two grid lines intersected over the endothelial glycocalyx.
